# Relationship Between Location of Cell Transplantation and Recovery for Intracerebral Stem Cell Transplantation for Chronic Traumatic Brain Injury: *Post-hoc* Analysis of STEMTRA Trial

**DOI:** 10.1089/neur.2024.0130

**Published:** 2025-01-28

**Authors:** Masahito Kawabori, Yasuaki Karasawa, Jun Suenaga, Hajime Nakamura, Hideaki Imai, Takao Yasuhara, Naoki Tani, Tatsuya Sasaki, Takashi Kawasaki, Kenta Totsuka, Dai Chida, Yoichi M. Ito, Tetsuya Yamamoto, Isao Date, Shota Tanaka, Haruhiko Kishima, Miki Fujimura

**Affiliations:** ^1^ Department of Neurosurgery, Hokkaido University Graduate School of Medicine, Sapporo, Japan.; ^2^ Department of Neurosurgery, Graduate School of Medicine, The University of Tokyo, Tokyo, Japan.; ^3^ Department of Neurosurgery, Yokohama City University School of Medicine, Yokohama, Japan.; ^4^ Department of Neurosurgery, Osaka University Graduate School of Medicine, Osaka, Japan.; ^5^ Department of Neurosurgery, JCHO Tokyo Shinjuku Medical Center, Tokyo, Japan.; ^6^ Department of Neurological Surgery, Okayama University Graduate School of Medicine, Okayama University Hospital, Okayama, Japan.; ^7^ Micron, Inc., Tokyo, Japan.; ^8^ SanBio, Inc., Tokyo, Japan.; ^9^ Biostatistics Division, Institute of Health Science Innovation for Medical Care, Hokkaido University Hospital, Sapporo, Japan.

**Keywords:** chronic, intracerebral transplantation, recovery, stem cell, traumatic brain injury

## Abstract

Traumatic brain injury is a world-leading cause of disability. Current treatments are not sufficient to promote neurological recovery. Intracerebral transplantation of allogeneic mesenchymal stem cells, specifically SB623, has shown promise in achieving better neurological recovery compared with a sham surgery group in the STEMTRA trial. However, the optimal location for cell transplantation remains unclear, as transplanted lesions vary between patients. This study aimed to explore the relationship between functional recovery and the location of transplanted lesions. This study included all Japanese subjects from the STEMTRA trial who were assigned to the cell transplantation group. Functional recovery was assessed by the difference in Fugl–Meyer Motor Scale (FMMS) scores between the screening period and 24 or 48 weeks post-transplantation. An FMMS score improvement of >8 was defined as an improved group. Lesions responsible for motor deficits were categorized into three groups: motor cortex (Cortex), deep white matter (DWM), or both (Cortex and DWM). Data on the 15 transplanted sites per patient were obtained from surgical navigation software, and the distance from the damaged area to the transplanted sites was calculated. Twelve patients were included in this *post-hoc* analysis. No patients in the 2.5 × 10^6^ cells group showed improvement and were therefore excluded from further analysis. Five patients were categorized into the Cortex group and four into the DWM group. The distance between the transplanted site and the injury point ranged from 0 to 39 mm. A moderate to strong trend of correlations was observed, suggesting that a shorter distance is preferable for the motor cortex group, while a greater distance is preferable for the DWM group. The optimal site for stem cell transplantation may be different from the damaged site of the patient; however, a further large number cohort is necessary to elucidate this hypothesis.

## Background and Objectives

Traumatic brain injury (TBI) is a global health problem, affecting more than 27 million cases worldwide each year.^1^ While advances in acute clinical care have resulted in improved rates of post-TBI survival,^2,3^ effective therapies for restoring neurological deficit are scarce. This leaves more than 50 million patients with long-term disabilities,^1^ emphasizing the need for the development of innovative treatment methods to restore neurological deficits.^4^

SB623 cells are adult bone-marrow-derived cells that have been transiently transfected with a plasmid construct encoding the intracellular domain of human Notch-1. Our previous double-blind, randomized, surgical sham-controlled, phase 2 stem cell therapy for TBI (STEMTRA) trial (NCT02416492) demonstrated that intracerebral SB623 transplantation facilitated significant motor recovery in patients with TBI, who suffer neurological deficit for more than 1 year.^5,6^ This study revealed that intracerebral transplantation of 5.0 or 10.0 × 10^6^ mesenchymal stem cells resulted in significant functional recovery compared with the control group, offering a potential avenue of hope for chronically handicapped patients with TBI.

In this clinical trial, the location for cell transplantations was determined according to the following rules based on the previous experiences in stroke trials^7^; each of three different trajectories comprised five cell deposit targets spaced 5–6 mm apart; the lesions should be set adjacent to the damaged area responsible for motor deficits based on the patient’s own neuroanatomy. Thus, each neurosurgeon determined the locations of cell transplantation where they believed to be optimal. Consequently, there was a variety of transplantation locations set for each patient. Although the overall result was promising, showing significant recoveries in the cell transplantation group, the ideal transplantation location for each patient has not been determined. With a view for better clinical application, identifying the optimal location for cell transplantation is crucial to maximize the efficacy of the surgical procedure. In this *post-hoc* analysis, we retrospectively analyzed the relationship between the location of cell transplantation and the extent of neurological recovery for Japanese subjects enrolled in the STEMTRA trial to elucidate the fundamental principle for cell transplantation locations.

## Methods

This study retrospectively collected the pre-surgical stereotactic transplantation plan in the neuro-navigation software in five Japanese sites that participated in the STEMTRA trial: Hokkaido University Hospital, University of Tokyo Hospital, Yokohama City University Hospital, Osaka University Hospital, and Okayama University Hospital. The study was approved by the institutional review board at Hokkaido University Hospital (approval number 019–0217), and informed consent was obtained using an opt-out method.

### Patients

This study included all Japanese subjects who were assigned to the SB623 transplantation group.^5,6^ Briefly, the major inclusion criteria of the patients were: aged 18–75; moderate or severe TBI (Glasgow Outcome Scale-Extended [GOS-E] scores of 3–6) who were at least 12 months post-injury; and neurological motor deficit substantially due to focal cerebral injury observed on magnetic resonance imaging (MRI).

### STEMTRA trial procedures

The detailed procedures have been previously reported.^5,6,8,9^ In brief, neuro-navigation software (Medtronic Stealth Cranial software, Hokkaido University Hospital, University of Tokyo Hospital, and Okayama University Hospital; BrainLab iStereotaxy software, Osaka University Hospital; Leksell SurgiPlan, Yokohama City University Hospital) were used to plan the entry point and the three implantation trajectories before the surgery. Three needle tracks are determined based on the patient’s preoperative MRI with trajectories to surround the responsive lesion. Each trajectory comprised five different cell deposits spaced 5 mm apart. As a result, 15 different cell deposits were performed for each patient. Each patient received 2.5 × 10^6^, 5.0 × 10^6^, and 10.0 × 10^6^ cells, with each deposit comprising 1.7 × 10^5^, 3.3 × 10^5^, and 6.7 × 10^5^ cells, respectively. On the day of surgery, patients were given local anesthesia and mild sedation, fitted with a Leksell stereotactic frame (Elekta Instruments, Stockholm, Sweden), and underwent a head computed tomography (CT)/MRI to overlay the coordinates for the targets and entry points. A single 15 mm burr hole was made at the coordinates using a craniotome. One hundred microliters of cell suspension were delivered per trajectory, and 20 µL were implanted for each deposit at a rate of 10 µL/min. Patients were observed in the recovery ward until they were fully awake and physiologically stable, after which they were returned to a neurosurgical ward. Functional assessments were conducted by neurologists, physiatrists, and physical therapists who were blind to patient treatment throughout the clinical trial.

### Assessment of injury and transplantation location

The focal cerebral injuries presumed to be the cause of motor deficits were defined by a neurosurgeon who determined the targets for its patients. Subsequently, it was assessed and approved by all Japanese neurosurgeons who participated in STEMTRA. The lesions were classified into three different groups: motor cortex (Cortex group), deep white matter (DWM group), or both (Cortex and DWM group). The Cortex group is defined when the responsible lesion of motor deficit is located on the surface of the motor cortex, whereas DWM is defined when the responsible lesion is located in the DWM, mostly at the posterior horn of the internal capsule and corona radiata. The Cortex and DWM group is defined when the patients’ brain damage responsible for the motor deficit is located in both areas. The preoperative stereotactic coordinates for 5 implantation sites × 3 trajectories were collected from the responsible neurosurgeons. Screen captures of navigation software showing axial, sagittal, coronal plane, and probe view plane, which represents the perpendicular view of the trajectory needle representing all transplanted sites, were obtained. Multiple slices of axial images showing three-dimensional focal injury were transcribed to the probe view for further analysis. Subsequently, the distance between the injured area and the 15 transplanted deposits was calculated. When the transplanted deposit is not in the same probe view plane, trigonometric functions to the nearest injury site were used to calculate the distance, and if the transplantation site was located within the target lesion, the estimated distance was set as 0 mm.

### Assessment of efficacy

The efficacy was evaluated according to the previously reported primary end-point, which is calculated by the difference of the Fugl–Meyer Motor Scale (FMMS) between the presurgical assessment and 24 weeks after transplantation (ΔFMMS 24 W). In addition, presurgical and 48 weeks after cell transplantation (ΔFMMS 48 W) were also evaluated. The patients who showed 8 points or more recovery in ΔFMMS 24 W were defined as the improved group, whereas patients who showed 7 points or less recovery were defined as the non-improved group.

### Statistical analysis

Data transcription and assessments were performed in a blinded manner. Statistical analyses were performed using GraphPad Prism (Ver. 10.2.0, Boston, MA, USA). Correlation of functional recovery and the transplanted distance from the transplanted site to the responsible lesion was conducted using simple linear regression analysis. Probability values of *p* < 0.05 were considered statistically significant, and an *R*^2^ value of 0.6 or higher was interpreted as indicating a strong correlation.

## Results

### Patient background

Thirteen patients with chronic motor deficit due to TBI were included in this analysis. However, surgical data of a single patient were not obtained due to the loss of surgical record and were excluded from the further analysis. Subsequently, 12 patients were included in this study (Table 1). The patients were all male, with ages ranging from 20 to 65 years old. The time since the injury ranged between 16 and 242 months. Among them, three patients (25%) received 2.5 × 10^6^ cells, four patients (33%) received 5.0 × 10^6^ cells, and five patients (42%) received 10.0 × 10^6^ cells. The average age of the patients, GOS-E, and time since injury were not significantly different between the cell groups. However, the number of patients classified into the improved group defer according to the number of cells transplanted; 0/3 (0%) for the 2.5 × 10^6^ cell group, 2/4 (50%) for the 5.0 × 10^6^ cell group, and 2/5 (40%) for 10.0 × 10^6^ cell group. This result was consistent with the previous report that the 2.5 × 10^6^ cell group did not show statistically significant recovery compared with the sham surgery in the STEMTRA study, in which cell numbers rather than transplantation distance were considered responsible for recovery, and these patients were excluded for further analysis.

**Table 1.** Patient Demographics

Transplanted cells	2.5 M	2.5 M	2.5 M	5 M	5 M	5 M	5 M	10 M	10 M	10 M	10 M	10 M
Age	23	41	65	29	29	31	34	20	36	41	43	49
Sex	Male	Male	Male	Male	Male	Male	Male	Male	Male	Male	Male	Male
Time since injury (month)	142	242	69	111	139	84	18	55	118	16	142	102
GOS-E	4	4	4	4	4	4	4	4	6	4	4	5
FMMS-UE												
Baseline	35	36	44	26	11	30	12	60	26	8	40	17
Δ24 W	0	−5	3	4	13	17	0	−4	0	24	14	4
Δ48 W	−4	0	6	0	11	18	0	−1	1	12	15	13
FMMS-LE												
Baseline	19	25	17	8	14	19	18	23	19	16	21	25
Δ24 W	5	1	1	1	5	2	0	2	4	10	1	2
Δ48 W	3	2	1	2	−1	2	−1	2	3	9	2	−1
DFMMS												
Δ24 W	5	−4	4	5	18	19	0	−2	4	34	15	6
Δ48 W	−1	2	7	2	10	20	−1	1	4	21	17	12
Lesion associated with motor deficit	DWM	DWM	Motor cortex	Motor cortex	DWM	Motor cortex	DWM	DWM	DWM	Motor cortex	DWM	Motor cortex

DWM, deep white matter; GOS-E, Glasgow Outcome Scale-Extended; FMMS, Fugl–Meyer Motor Scale; LE, lower extremity; M = × 10^6^; MRI, magnetic resonance imaging; UE, upper extremity.

Regarding the lesions associated with motor deficit, four patients (44%) were classified into the Cortex group and five (56%) were classified into the DWM group, and there was no patient classified in the Cortex and DWM group.

The relationship of functional recovery and the distance of transplantation.

**FIG. 1.**
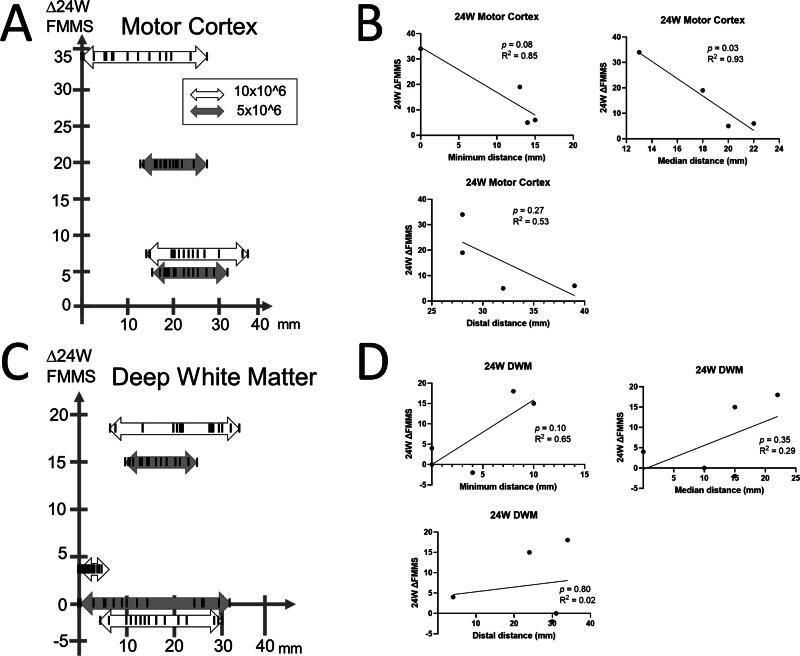
Relationship of distance between site of cell transplantation and functional recovery of 24 weeks after transplantation. **(A)** Functional recovery at 24 weeks and all transplantation sites are shown for a patient with motor cortex damage. The black perpendicular line in each arrow represents the distance of all 15 transplantation sites. **(B)** The correlation between recovery and transplantation distance was evaluated for three deposits: the closest deposit (minimum distance), the median deposit (median distance), and the farthest deposit (distal distance). For minimum and median distances, closer cell transplantation sites were associated with improved functional recovery. **(C)** Functional recovery at 24 weeks and all transplanted sites are shown for patients with deep white matter damage. **(D)** Greater functional recovery was observed with farther transplantation sites for minimum and median distances.

**FIG. 2.**
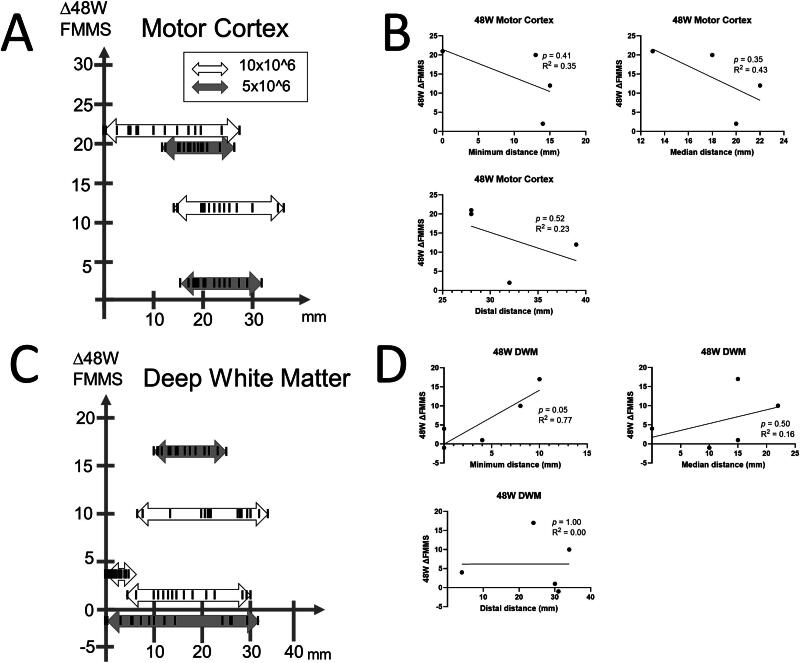
Relationship of distance between site of cell transplantation and functional recovery of 48 weeks after transplantation. **(A)** Functional recovery at 48 weeks and all transplanted sites are shown for patients with motor cortex damage. **(B)** Minimum distance and median distance presented that closer cell transplantation showed better functional recovery. **(C)** Functional recovery at 24 weeks and all transplantation sites are shown for a patient with deep white matter damage. **(D)** Farther cell transplantation showed better recovery for minimum distance.

**FIG. 3.**
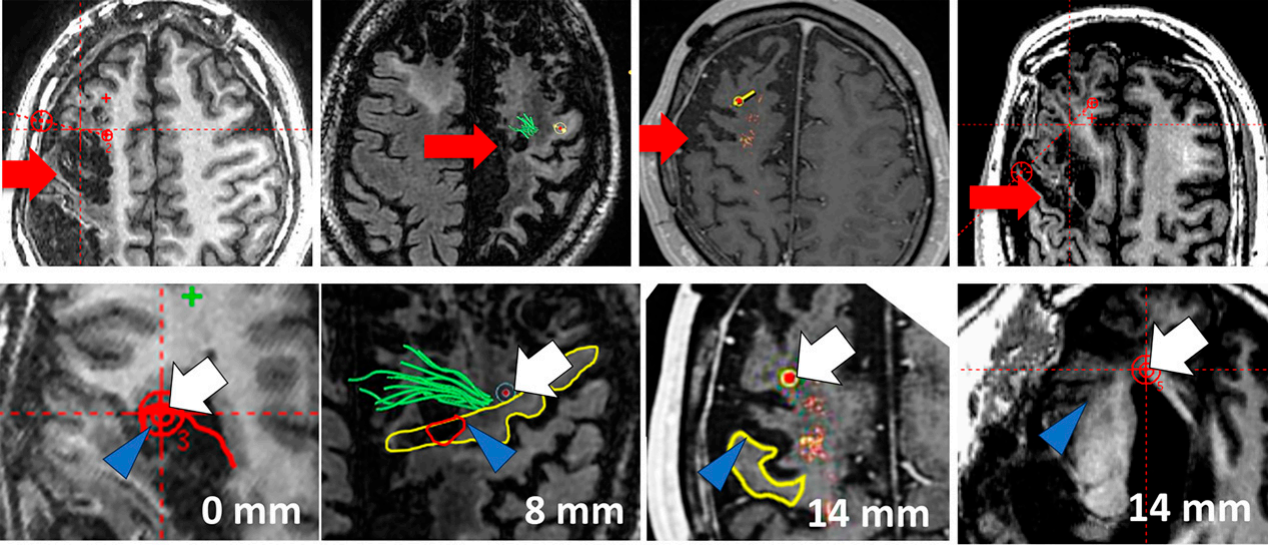
Representative figures of the patients with motor cortex damage. (Upper row) MRI axial image of the damaged area, where the red arrow indicates the actual damaged site. (Lower row) Prove eye views of the closest point between cell transplantation and the damaged area. The white arrow indicates the transplanted site, and the blue arrow indicates the closest point of the damaged area. The yellow area indicates the motor cortex, and the red circle indicates the responsible damaged area. MRI, magnetic resonance imaging.

**FIG. 4.**
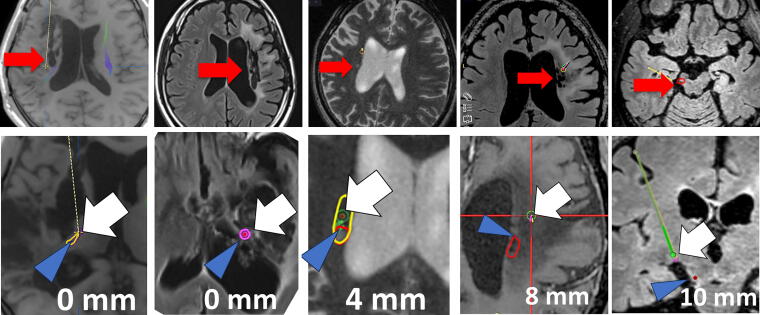
Representative figures of the patients with deep white matter damage. (Upper row) MRI axial image of the damaged area, where the red arrow indicates the actual damaged site. (Lower row) Probe eye views of the closest point between cell transplantation and the damaged area. The white arrow indicates the transplanted site, and the blue arrow indicates the closest point of the damaged area. The yellow area indicates the motor cortex, and the red circle indicates the responsible damaged area. MRI, magnetic resonance imaging.

The distance of 15 cell transplanted sites (deposits) to the damaged lesion in nine patients ranged between 0 and 39 mm (Figs. 1A, C and 2A, C). The distance between the closest deposit and the farthest deposit in each patient varies as shortest patient received 15 deposits within 4 mm, while the longest patient received that within 31 mm. Representative figures of each patient in the Cortex group and DWM groups with the closest deposit are shown in Figures 3 and 4. Subsequently, three deposits—the closest deposit (minimum distance), median deposit (median distance), and the farthest deposit (distal distance)—for each patient were evaluated to elucidate relationship with the function recovery. In the Cortex group, we found an interesting trend that a shorter distance between the median deposit and damaged area was correlated with better functional recovery in ΔFMMS 24 W (*p* = 0.03, *R*^2^ = 0.93). This trend was also observed for the distance between the closest deposit and damaged area (*p* = 0.08, *R*^2^ = 0.85) (Fig. 1B). In contrast, the DWM group showed opposite results that better recoveries were seen in the patient whose closest deposit were further from the injury (*p* = 0.10, *R*^2^ = 0.65) (Fig. 1D). There was no correlation between the recovery and the most distal deposit point. Regarding the ΔFMMS 48 W result (Fig. 2A, C), these above-mentioned trends were also observed in both groups: shorter distance of closest and median deposit was preferable for the Cortex group (*p* = 0.41, *R*^2^ = 0.35, *p* = 0.35, *R*^2^ = 0.43, respectively) (Fig. 2B), while farther distance of closest deposit was preferable for the DWM group (*p* = 0.05, *R*^2^ = 0.77) (Fig. 2D). Of note, patients who showed unfavorable recovery in the DWM group were transplanted almost into the damaged lesion or very close to the lateral ventricle, where cell survival or engraftment may be disturbed.

## Discussion

In this study, the authors were able to identify two key findings. First, the group of Japanese patients receiving 2.5 × 10^6^ cells demonstrated unsuccessful functional recovery, consistent with the original assessment across other racial groups. Second, there were divergent results concerning functional recovery and the distance between the transplanted site and the damaged area, depending on the nature of the brain lesion. Patients with motor cortex damage exhibited better recovery when the cells were transplanted near the damaged area, whereas those with DWM damage showed a preference for transplantation in a distant area.

There have been many cell transplantation routes examined in clinical trials, including intracerebral, intravenous (IV), and intraarterial (IA) transplantation. IV transplantation offers the advantage of being the least invasive. However, despite its efficacy in pre-clinical models, only small amounts of cells are typically found in the damaged brain, with most cells becoming trapped in the lung and liver.^10^ The IA approach is considered superior to IV administration in delivering more cells to the affected brain. However, recent reports have indicated that effective cell engraftment is not as expected.^11–13^ Because cells transplanted intravenously or intra-arterially are unable to stay in the brain for long periods, these methods are considered useful in the acute phase of the disease, where regulating inflammation is one of the most important aspects of treatment.^11–14^ Intracerebral transplantation enables the transfer of a sufficient amount of cells to the brain but carries the risk of additional brain damage with the transplantation procedures. In this circumstance, Hess et al. proposed that IV or IA transplantation is preferred in the acute phase, whereas intracerebral transplantation with or without a bioscaffold is preferred after the sub-acute phase, where the reorganization of brain structure is required as a mechanism of stem cell action.^15^ This principle is considered reasonable, and many clinical trials follow the principle of timing and transplantation route, including this study.^16–18^

The data on the distance from the injury to the transplanted site are quite important to maximize the therapeutic result. However, the pre-clinical experiment is quite challenging because the rodent model is too small to accurately evaluate the distance of cell transplantation and the damaged lesion. To the best of our knowledge, this is the first report focusing on this issue. We were able to elucidate interesting results regarding the distance of cell transplantation in different patient backgrounds, in which a shorter distance was preferable for patients with motor cortex damage, while a farther distance was preferable for patients with DWM damage. Our finding in the motor cortex is understandable, as better function is achieved when the cell is transplanted closer to the damaged motor cortex. We and others have reported that transient FLAIR (Fluid Attenuated Inversion Recovery) high signals of MRI are observed at the transplanted lesion within a few months after transplantation, and this signal correlates with positive recovery.^8,19,20^ Kawabori et al. reported that the size of the FLAIR high signal ranges between 1 and 3 cm. Considering that the FLAIR high signal is a consequence of neurotrophic factors released from the stem cells, the distance of cell transplantation should be within this range. However, the reason for our opposite findings in DWM remains unknown, as closer cell transplantation was inferior to farther cell transplantation. One possible explanation is that the transplanted site in these patients is too close to the damaged lesion, resulting in insufficient brain condition, including a lack of sufficient oxygen supply, for efficient cell engraftment. Since lead placement therapy studies have reported that post-operative CT scans revealed a radial error of 1.2 ± 0.5 mm from the proposed target in deep brain stimulation surgery,^21^ misplacement of cells into the ventricle or surrounding necrotic tissue could also be considered. This trial required three independent needle insertions into the atrophied brain, which increases the risk of brain shift compared to ordinary lead placement therapy. In the future, the cells should be transplanted into an area where a certain margin can be obtained for patients with DWM brain damage.

There are many limitations to acknowledge in this trial. First, the number of subjects is quite small, making it difficult to obtain definitive results. We were unable to obtain data from patients in the United States and Ukraine due to the contractual constraints of the STEMTRA trial. Further data acquisition is strongly required to comprehensively evaluate our results. Second, the data examined in this trial were obtained from pre-surgical plans and not from postoperative data. As mentioned above, stereotactic surgery potentially carries the risk of malpositioning of the needle due to brain shift. Data from the transplantation of labeled cells will be necessary to elucidate the accuracy of the procedure. Third, the area responsible for the motor functional deficit may not be accurately identified. Although experienced neurosurgeons involved in this trial agreed on the responsible lesions, objective methods such as the discontinuation of diffusion tensor imaging may help clarify the treatment targets. Fourth, we calculated the direct distance between the lesion and the transplanted site, without considering the gyrus, especially in the motor cortex. The actual distance from the lesion to the transplanted site may differ when accounting for the presence of gyrus.

## Conclusion

The optimal site for stem cell transplantation may vary depending on the location of the patient’s brain damage. When the patient’s brain damage is located in the motor cortex, cell transplantation may be considered to be near the affected area. Conversely, if the brain damage is situated in the DWM, the location of cell transplantation should be selected carefully to avoid proximity to the damaged lesion. Instead, it should be chosen based on where cell engraftment is highly secure.

## Abbreviations Used

CT: Computed tomography
FLAIR: Fluid Attenuated Inversion Recovery
FMMS: Fugl-Meyer Motor Scale
GOS-E: Glasgow Outcome Scale-Extended
MRI: Magnetic resonance imaging
TBI: traumatic brain injury
